# Combined training improves functional capacity, fatigue, and quality of life in individuals with multiple sclerosis: a systematic review and meta-analysis

**DOI:** 10.1186/s12883-025-04548-z

**Published:** 2025-12-03

**Authors:** Fereshteh Kazemi Pakdel, Ahmad Kazemi Pakdel, Hamed Zarei, Ali Brian

**Affiliations:** 1 Corrective Exercises and Sports Injury Department, Faculty of Physical Education & sport sciences, University of Guilan, Rasht, 4199613776 Iran; 2 Department of Physical Education, University of South Carolina, Columbia, SC USA

**Keywords:** Multiple sclerosis, Combined training, Physical outcomes

## Abstract

**Introduction:**

Multiple Sclerosis (MS) severely affects strength, coordination, gait, and balance, leading to significant challenges for individuals. While exercise is known to benefit MS management, most studies focus on single modalities, leaving a gap in understanding the effects of combined training (CT) interventions. This systematic review aims to address this gap by examining the impact of diverse CT interventions on muscle strength, balance, gait speed, endurance, fatigue, and quality of life in people with MS (PwMS). The findings will provide valuable insights for enhancing rehabilitation strategies tailored to the multifaceted needs of PwMS.

**Methods:**

Primary sources were gathered from eight databases: PubMed, SCOPUS, Embase, EBSCO, CENTRAL (Cochrane Central Register of Controlled Trials), CINAHL, PEDro, and Web of Science. The search timeframe ranged from the beginning of the study period until July 25, 2025. Quality assessment scores for the eligible studies were determined using the Physiotherapy Evidence Database (PEDro). Standardized mean differences (SMD), and 95% confidence intervals (CIs) were computed using either random or fixed models for the outcomes assessed.

**Results:**

Following the screening process, 20 studies involving a total of 577 participants were included in the systematic review. The results of the meta-analysis showed that CT improved lower limb muscle strength (1.04 [95% CI: 0.59–1.49], *p* = 0.001), balance (1.20 [95% CI: 0.76–1.64], *p* = 0.001), gait speed (0.77 [95% CI: 0.35–1.18], *p* = 0.001), gait endurance (0.96 [95% CI: 0.46–1.46], *p* = 0.001), and quality of life (0.69 [95% CI: 0.43–0.96], *p* = 0.001). Also, the results indicated that CT decreased fatigue (0.82 [95% CI: 0.57–1.08], *p* = 0.001).

**Conclusion:**

The systematic review and meta-analysis provide compelling evidence that CT interventions positively influence physical and functional outcomes in PwMS. Specifically, the results demonstrate significant improvements in muscle strength, balance, gait speed, gait endurance, and quality of life, alongside a notable reduction in fatigue. These findings underscore the potential of CT as an effective rehabilitation and management strategy for PwMS, suggesting that incorporating such interventions into clinical practice could enhance patient outcomes. By addressing key functional impairments and improving overall well-being, CT can play a crucial role in optimizing the quality of life for individuals living with multiple sclerosis, ultimately leading to more effective and holistic management of the condition.

**Registry number:**

CRD420251111193

**Supplementary Information:**

The online version contains supplementary material available at 10.1186/s12883-025-04548-z.

## Introduction

Multiple Sclerosis (MS) is an autoimmune disorder that leads to a chronic and progressive condition impacting the Central Nervous System (CNS) [1]. A hallmark of MS is the degeneration of myelin, the protective covering surrounding the axons of nerve cells, which leads to the development of characteristic plaques [2]. Multiple Sclerosis is classified into four main types: benign, relapsing-remitting, primary progressive, and secondary progressive [3]. The condition is usually diagnosed in individuals during their prime working years, particularly between the ages of 20 and 40 years.

Lesions associated with MS present a range of symptoms in patients, which can significantly impact their daily lives. The specific nature and severity of these symptoms are influenced by the location and extent of the lesions [4]. Current clinical manifestations of MS encompass impairments in motor, sensory, visual, and genitourinary functions [5]. Regarding mobility, MS contributes to diminished strength, coordination, gait, and balance [6]. These difficulties can lead to considerable neurological disabilities, physical deconditioning, worsening of symptoms, decreased quality of life (QoL), and a progressive loss of independence for the substantial population affected by this condition [7].

Disease-modifying therapies are the main strategy for slowing the progression of MS. Additionally, rehabilitation strategies, especially exercise training programs, act as a complementary approach to address neurological disabilities and manage challenges related to physical deconditioning, worsening symptoms, reduced QoL, and loss of independence over time in individuals with MS [8–11]. Exercise training programs are generally safe, associated with minimal adverse effects, and may help lower the risk of relapses in people with MS (PwMS). These programs can encompass a variety of modalities, including resistance training [12], aerobic exercises [13], and balance activities [14]. The exercises in question are unidimensional, focusing on a single type of training regimen throughout the course. Recent research has explored the effects of combining different exercises on various aspects of motor performance, balance [15], muscle strength, gait [16], fatigue, and overall health-related quality of life in PwMS [17]. The findings of these studies indicate that combined training exercises have a substantial impact, leading to significant improvements in the specified variables.

Combined training (CT) involves the integration of two distinct types of exercises that are performed concurrently within a single training session [18]. This simultaneous performance of different exercise types enhances overall physical fitness by targeting various health components, including cardiovascular endurance, muscle strength, flexibility, and balance [19]. The efficiency of CT is particularly advantageous, as it enables individuals to work on multiple fitness aspects without necessitating separate training sessions [20].

A synthesis of evidence from systematic review has shown that combined balance and strength training are effective methods for improving postural balance following a stroke [21]. Additionally, a separate meta-analysis assessed the effects of aerobic, resistance, and combined exercise training on health-related quality of life (HRQOL) in PwMS. The results of this review indicated that exercise training is clinically effective in enhancing overall HRQOL in this population [13]. Furthermore, another meta-analysis explored the impact of combined strength and endurance training on fatigue in patients with multiple sclerosis. The findings revealed that CT can be an effective strategy for helping PwMS manage their fatigue [22]. However, this study was limited to a single outcome and specifically focused on the combination of strength and endurance training.

Multiple Sclerosis presents significant challenges to individuals, severely affecting muscle strength, balance, gait speed, and overall quality of life. While numerous studies have explored the benefits of exercise, the majority have concentrated on single modalities, leaving a substantial gap in understanding the multifaceted effects of CT interventions. This study aims to address this gap by comprehensively examining the impact of a diverse range of CT on critical variables, including muscle strength, balance, gait endurance, fatigue, and quality of life in PwMS. By integrating both aerobic and resistance training, this research offers a novel approach that not only contributes to the existing body of knowledge but also provides therapists with a holistic framework for developing effective, individualized rehabilitation strategies. Systematic reviews and meta-analyses have underscored the efficacy of CT in improving HRQOL and managing fatigue among PwMS [13, 22]; however, previous studies have often focused on specific outcomes or singular combinations of exercise types, limiting the understanding of the comprehensive benefits of CT modalities. By exploring a wider range of CT interventions, this study seeks to provide deeper insights into how these approaches can enhance overall functional outcomes and quality of life for PwMS, ultimately fostering greater independence and well-being. The findings are expected to empower both therapists and patients, facilitating the development of more effective rehabilitation protocols tailored to the unique needs of individuals with MS.

## Method

This systematic review followed the guidelines set forth by the Preferred Reporting Items for Systematic Reviews and Meta-Analyses (PRISMA) [23]. The protocol for the review has been registered in the PROSPERO database under the identification number CRD420251111193.

### Search strategy

Primary sources were collected from eight databases: PubMed, SCOPUS, Embase, EBSCO, CENTRAL (Cochrane Central Register of Controlled Trials), CINAHL, PEDro, and Web of Science. The search timeframe ranged from the beginning of the study period until July 25, 2025, with no restrictions on publication dates. Initially, Medical Subject Headings (MeSH) terms were employed to guide the selection of keywords, which were subsequently refined to ensure a thorough coverage of relevant studies. To expand our search further, we utilized Google Scholar, enabling us to incorporate articles from a variety of databases. The selection process involved independent reviews by two researchers who screened titles and abstracts for relevance based on predefined inclusion criteria. Following this initial screening, the full texts of potentially eligible studies were assessed by the same reviewers. Any discrepancies between the reviewers’ assessments were resolved through discussion and consensus. Additionally, after completing the selection process, we reviewed the references of the included studies to identify any potentially missed citations, ensuring a comprehensive evaluation of relevant literature. The electronic databases were searched using combinations of the following keyword groups: (1) “multiple sclerosis” OR “MS” OR “degenerative nerve disease” OR “neurological disorder” OR “neuroinflammation” OR “demyelination” OR “autoimmune disease” OR “relapsing-remitting MS” OR “Primary progressive” OR “secondary progressive MS”; AND (2) “combined training” OR “concurrent training” OR “cross-training” OR “training modalities” OR “holistic training” OR “mixed training methods”. The “AND” operator was utilized to link the three groups of keywords, whereas the “OR” operator was applied within each individual keyword group.

### Eligibility criteria

The inclusion criteria were defined as follows: (1) Population: people with multiple sclerosis; (2) Intervention: combined training program; (3) Comparator: Comparison of combined training program with a non-training control group; (4) Outcomes: muscles strength, balance, gait speed, gait endurance, fatigue, quality of life; (5) Study Designs: Randomized controlled trials; (6) Peer-reviewed articles published in Persian or English. Two independent researchers (X.X. and X.X.) performed the search and screened titles and abstracts according to the predetermined criteria. Any discrepancies were addressed through discussion.

### Data extraction

Researchers (X.X. and X.X.) independently extracted data from the studies using various metrics, which included the first author’s name, study design, participant characteristics (such as sample size, age range or mean age with standard deviation (SD), and sex distribution), outcome measures, measurement methods and units, as well as details about the training programs and their specific features (see Table 1).

**Table 1:** General description of the samples included in the individual studies

Source, year	Study design	Sample size	Sex	Age [years]	Groups	Participants characteristics	Intervention	Training characteristics= Frequency (time a week)/Duration (week)	Outcomes measure
Abaspour et al. 2022 [27]	RCT, Two arms	16	F	EXP = 33.50 ± 6.37 CON = 36.75 ± 6.80	EXP = 8 CON = 8	People with multiple sclerosis. EDSS < 5	EXP = Rhythmic aerobic and resistance training by bodyweight, Theraband, and TRX CON = Routine daily activities	3/8	- Muscles strength (kg) - Gait endurance (meter) - Gait speed (sec)
Monireh et al. 2013 [28]	RCT, Two arms	20	F	EXP = 36.10 ± 2.92 CON = 33.00 ± 5.87	EXP = 10 CON = 10	People with multiple sclerosis. EDSS ≤ 5	EXP = Resistance and aerobic training CON = Routine daily activities	3/8	- Muscles strength (kg) - Balance (sec) - Gait speed (sec)
Broekmans et al. 2011 [29]	RCT, Two arms	26	F (17) M (9)	EXP = 48.7 ± 8.6 CON = 49.7 ± 11.3	EXP = 11 CON = 14	People with multiple sclerosis. EDSS = 4.3 ± 0.2	EXP = Resistance training with simultaneous electro-stimulation CON = Routine daily activities	3/20	- Muscles strength (kg) - Balance (sec) - Gait speed (sec) - Gait endurance (meter)
Callesen et al. 2020 [12]	RCT, Two arms	48	F (32) M (16)	EXP = 51.2 ± 10.6 CON = 50.3 ± 8.4	EXP = 28 CON = 20	People with multiple sclerosis. EDSS = 2.0–6.5	EXP = Balance and motor control training CON = Routine daily activities	3/20	- Muscles strength (kg) - Balance (sec) - Gait speed (sec) - Gait endurance (meter) - Fatigue (score)
Eftekhari et al. 2012 [30]	RCT, Two arms	24	F	EXP = 35.08 ± 6.89 CON = 33.75 ± 5.32	EXP = 12 CON = 12	People with multiple sclerosis. EDSS = 2.0–4.0	EXP = Resistance Training and Vibration CON = Routine daily activities	3/8	- Muscles strength (kg) - Balance (sec) - Gait speed (sec)
Monireh et al. 2012 [38]	RCT, Two arms	20	F	EXP = 34.55 ± 4.78 CON = 34.55 ± 4.78	EXP = 10 CON = 10	People with multiple sclerosis. EDSS = 2.95 ± 1.54	EXP = Resistance, aerobic and balance CON = Routine daily activities	3/6	- Balance (sec) - Gait speed (sec) - Gait endurance (meter)
Haghighi et. 2023 [31]	RCT, Two arms	14	F (8) M (6)	EXP = 32.00 ± 4.11 CON = 36.00 ± 4.86	EXP = 7 CON = 7	People with multiple sclerosis. EDSS = 2.95 ± 1.54	EXP = Resistance and aerobic training CON = Routine daily activities	3/8	- Muscles strength (kg) - Balance (sec) - Gait endurance (meter)
Abbaspoor et al., 2020 [32]	RCT, Two arms	16	F	EXP = 33.50 ± 6.37 CON = 36.75 ± 6.80	EXP = 8 CON = 8	People with multiple sclerosis. EDSS < 5	EXP = Rhythmic aerobic exercise, TRX suspension training, elastic band training, and bodyweight training CON = Routine daily activities	3/8	- Muscles strength (kg) - Gait speed (sec) - Gait endurance (meter)
Gutiérrez-Cruz et al. 2020 [33]	RCT, Two arms	26	F (16) M (10)	EXP = 40.7 ± 8.2 CON = 47.2 ± 9.8	EXP = 15 CON = 11	People with multiple sclerosis. EDSS < 6	EXP = Strength exercises and cognitive–motor tasks CON = Routine daily activities	3/24	- Muscles strength (kg) - Balance (sec) - Gait speed (sec)
Correale et al. 2021 [34]	RCT, Two arms	27	F	EXP = 40.7 ± 8.2 CON = 47.2 ± 9.8	EXP = 14 CON = 13	People with multiple sclerosis. EDSS < 4	EXP = Resistance and endurance training CON = Routine daily activities	2/12	- Muscles strength (kg) - Quality of life (score) - Fatigue (score)
Sangelaji et al. 2016 [35]	RCT, Two arms	20	F (12) M (8)	EXP = 33.91 ± 7.94 CON = 33.63 ± 6.92	EXP = 10 CON = 10	People with multiple sclerosis. EDSS < 5	EXP = Resistance and aerobic training CON = Routine daily activities	4/8	- Muscles strength (kg) - Balance (sec) - Gait speed (sec) - Gait endurance (meter)
Sangelaji et al. 2014 [39]	RCT, Two arms	59	F (38) M (21)	EXP = 33.05 ± 7.68 CON = 32.05 ± 6.35	EXP = 39 CON = 20	People with multiple sclerosis. EDSS < 4	EXP = Aerobic, strengthening, balancing and stretching exercises CON = Routine daily activities	3/10	- Quality of life (score) - Balance (score) - Gait endurance (meter) - Fatigue (score)
Ray et al. 2013 [43]	RCT, Two arms	21	F (16) M (5)	EXP = 50.9 ± 5.7 CON = 56.2 ± 8.8	EXP = 11 CON = 10	People with multiple sclerosis. EDSS ≤ 6.5	EXP = Combined (inspiratory and expiratory), progressive resistance respiratory muscle training CON = Routine daily activities	3/5	- Quality of life (score) - Fatigue (score)
Alvarenga-Filho et al. 2016 [44]	RCT, Two arms	18	F (13) M (5)	EXP = 41.1 ± 12.9 CON = 35.2 ± 7.6	EXP = 8 CON = 10	People with multiple sclerosis. EDSS ≤ 2	EXP = Pilates and aerobic exercises CON = Routine daily activities	3/12	- Fatigue (score)
Ozkul et al. 2020 [42]	RCT, Two arms	34	F (26) M (8)	EXP = 35.88 ± 9.74 CON = 36.76 ± 9.02	EXP = 17 CON = 17	People with multiple sclerosis. EDSS < 4	EXP = Pilates and aerobic exercises CON = Routine daily activities	3/8	- Quality of life (score) - Gait endurance (meter) - Fatigue (score)
Najafi et al. 2019 [40]	RCT, Two arms	56	F	EXP = 38.39 ± 4.59 CON = 36.36 ± 3.54	EXP = 28 CON = 28	People with multiple sclerosis. EDSS < 4.5	EXP = Stability exercises and specific to postural control CON = Routine daily activities	3/8	- Balance (sec)
Attar Sayyah et al. 2016 [45]	RCT, Two arms	37	F (22) M (15)	EXP = 34.53 ± 6.51 CON = 36.78 ± 4.93	EXP = 19 CON = 18	People with multiple sclerosis. EDSS = 1–5	EXP = Resistance and proprioceptive neuromuscular facilitation CON = Routine daily activities	3/8	- Quality of life (score) - Fatigue (score)
Grazioli et al. 2019 [41]	RCT, Two arms	20	F (15) M (5)	EXP = 45.91 ± 12.09 CON = 39.40 ± 10.26	EXP = 10 CON = 10	People with multiple sclerosis. EDSS = 2–5	EXP = Resistance and aerobic training CON = Routine daily activities	3/8	- Quality of life (score) - Balance (score) - Gait endurance (meter) - Fatigue (score) - Gait speed (sec)
Sayyah et al. 2016 [36]	RCT, Two arms	37	F (22) M (15)	EXP = 34.53 ± 6.51 CON = 36.78 ± 4.93	EXP = 19 CON = 18	People with multiple sclerosis. EDSS = 2–4	EXP = Resistance and proprioceptive neuromuscular facilitation CON = Routine daily activities	3/8	- Muscles strength (kg) - Balance (sec) - Gait speed (sec) - Gait endurance (meter)
Kordi et al. 2011 [37]	RCT, Two arms	38	F (24) M (14)	EXP = 31.63 ± 6.00 CON = 36.78 ± 4.93	EXP = 19 CON = 19	People with multiple sclerosis. EDSS = 0–4	EXP = Aerobic, strengthening, balancing and stretching exercises CON = Routine daily activities	3/8	- Muscles strength (kg) - Quality of life (score) - Balance (score)

*NR* Not reported, *SD* Standard deviation, *M* Male, *F* Female, *EXP* Experimental group, *CON* Control group, *RCT* randomized control tr

### Quality of evidence

Quality assessment scores for the eligible studies were determined using the Physiotherapy Evidence Database (PEDro). The total PEDro score, which ranges from 0 to 11, takes into account factors such as the reporting of statistical analysis and criteria for evaluating internal validity. Studies that scored between 7 and 11 were classified as having “high” methodological quality, those with scores of 5 to 6 were considered “fair,” and studies scoring ≤ 4 were categorized as “poor” [24].

### Statistical analyses

Heterogeneity was evaluated using the I² index, with the following thresholds: 0%–30% indicating no heterogeneity; 30%–50% indicating low heterogeneity; 50%–75% indicating moderate heterogeneity; and 75%–100% indicating high heterogeneity. In this study, both random and fixed-effects models were employed to address between-study heterogeneity. A random effects model was utilized when the I² value exceeded 50% [25]. Standardized mean differences (SMDs) and 95% confidence intervals (CIs) were calculated using either random or fixed-effect models for the outcomes, with a significance threshold set at *p* ≤ 0.05. Funnel plots and Egger’s tests were visually assessed to detect publication biases, with Egger’s test serving as a secondary analysis when *p* < 0.1. The trim-and-fill correction method was applied as necessary to mitigate potential publication biases [26]. Statistical analysis was conducted using Comprehensive Meta-Analysis version 2.0 (Biostat Inc, Englewood, New Jersey).

## Results

**Fig. 1:**
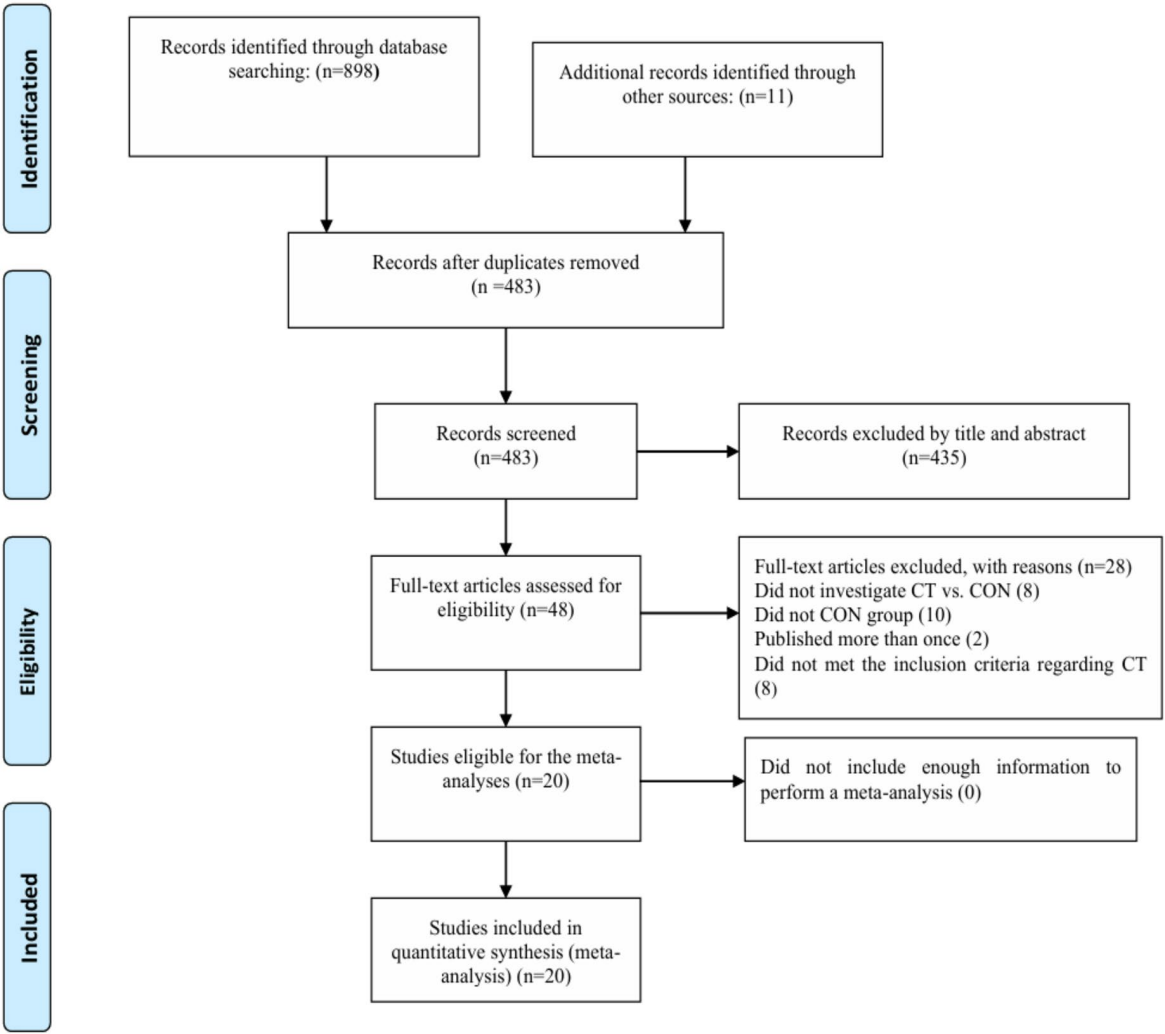
Flow diagram of systematic literature search. CON, control group; CT, combined training

A total of 898 potentially eligible studies were initially identified across eight databases. Additionally, 11 records were discovered through the ancestry method by screening reference lists. After eliminating 426 duplicate studies, 483 titles and abstracts were screened, resulting in the exclusion of 435 entries. The full texts of the remaining 48 studies were obtained for comprehensive evaluation. From these, 28 studies were excluded for failing to meet the eligibility criteria. No studies were excluded from the analysis due to insufficient information required for conducting a meta-analysis. Ultimately, 20 studies involving a total of 577 participants were included in the systematic review (see Fig. 1).

### Quality of evidence

According to the PEDro Scale, among the 20 studies that examining the effects of CT program on various outcomes of PwMS, 10 studies (50%) achieved high scores ranging from 7 to 11, while 10 studies (50%) fell within the fair score range of 5 to 6. Overall, the quality of the studies analyzed using the PEDro Scale was deemed adequate, indicating their reliability for inclusion in the present meta-analysis (see Supplemental Table).

## Meta-analysis

### Effect of combined training program on lower limb muscle strength

**Fig. 2:**
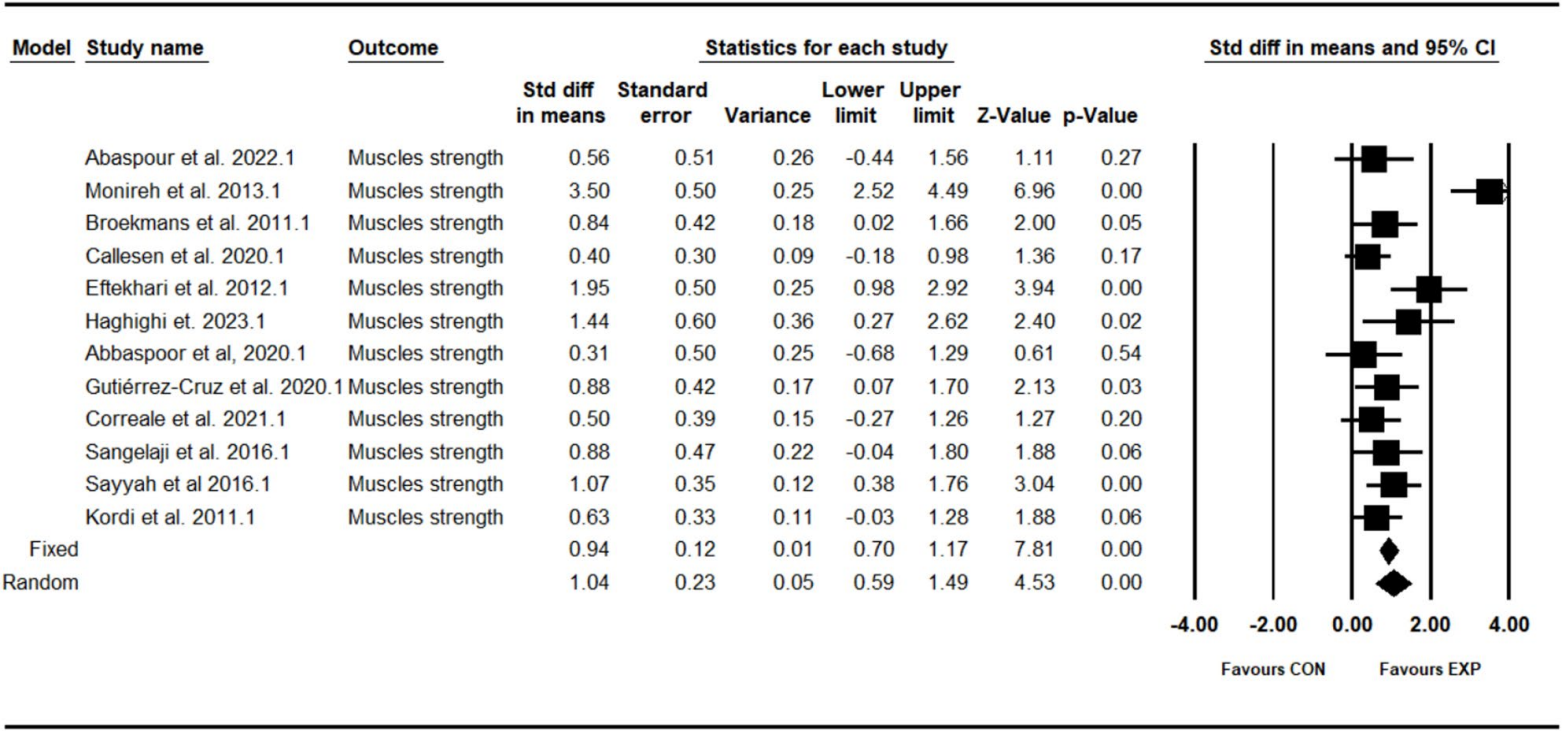
Forest plot of the combined training program versus control group analysis on muscle strength

The analysis included twelve intervention arms examining the effect of the CT program on the muscle strength [12, 27–37]. Overall, CT program demonstrated a significant improvement in the muscle strength (1.04 [95% CI: 0.59–1.49], *p* = 0.001), and moderate significant heterogeneity among the studies (I² = 71.51%, *p* = 0.001) (see Fig. 2). Both the visual assessment of funnel plots and the results from Egger’s test did not indicate any significant publication bias (*p* = 0.11). The trim-and-fill method identified one missing study on the right side of the mean; however, the results remained unchanged after correction (0.97 [95% CI: 0.65–1.50]).

### Effect of combined training program on balance

**Fig. 3:**
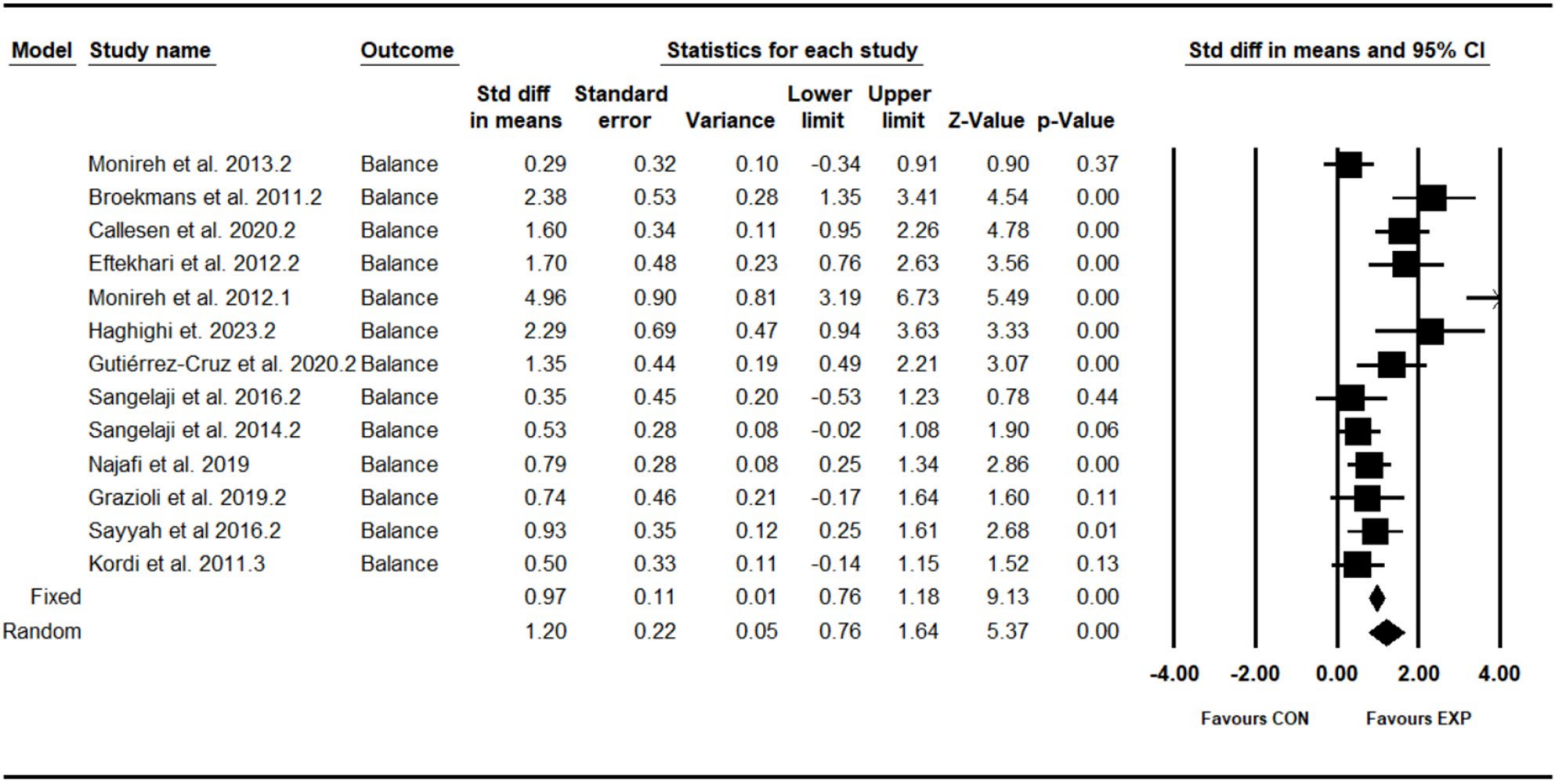
Forest plot of the combined training program versus control group analysis on balance

The analysis included thirteen intervention arms examining the effect of the CT program on the balance [12, 28, 30, 31, 33, 35–41]. Overall, CT program demonstrated a significant improvement in the balance (1.20 [95% CI: 0.76–1.64], *p* = 0.001), and moderate significant heterogeneity among the studies (I² = 75.39%, *p* = 0.001) (see Fig. 3). Both the visual assessment of funnel plots and the results from Egger’s test did not indicate any significant publication bias (*p* = 0.26). The trim-and-fill method identified three missing studies on the left side of the mean; however, the results remained unchanged after correction (0.81 [95% CI: 0.34–1.35]).

### Effect of combined training program on gait speed

**Fig. 4:**
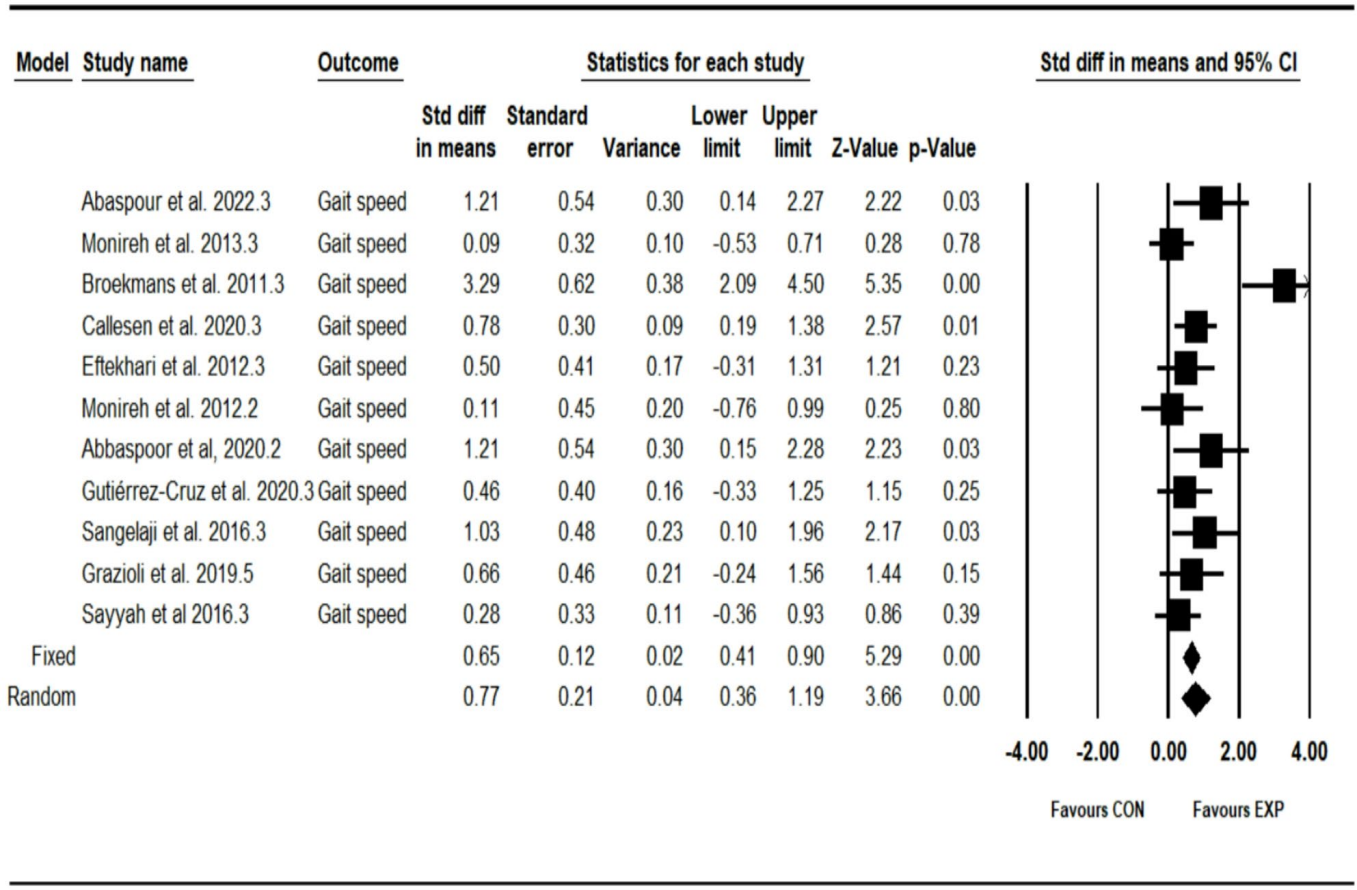
Forest plot of the combined training program versus control group analysis on gait speed

The analysis included eleven intervention arms examining the effect of the CT program on the gait speed [12, 27–30, 32, 33, 35, 36, 38, 41]. Overall, CT program demonstrated a significant improvement in the gait speed (0.77 [95% CI: 0.35–1.18], *p* = 0.001), and moderate significant heterogeneity among the studies (I² = 63.70%, *p* = 0.002) (see Fig. 4). Both the visual assessment of funnel plots and the results from Egger’s test did not indicate any significant publication bias (*p* = 0.36). The trim-and-fill method identified two missing studies on the left side of the mean; however, the results remained unchanged after correction (0.50 [95% CI: 0.04–1.03]).

### Effect of combined training program on gait endurance

**Fig. 5:**
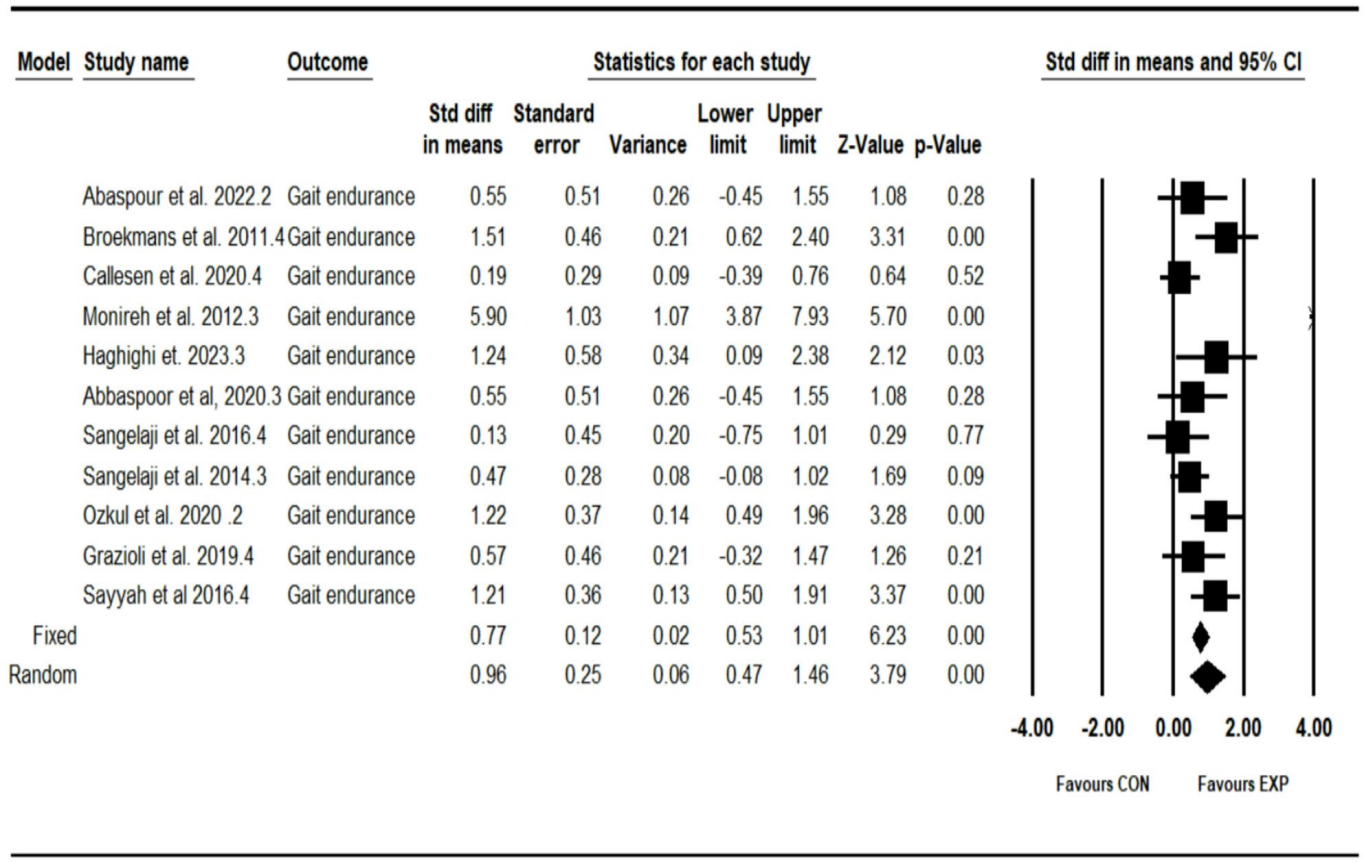
Forest plot of the combined training program versus control group analysis on gait endurance

The analysis included eleven intervention arms examining the effect of the CT program on the gait endurance [12, 27, 29, 31, 32, 35, 36, 38, 39, 41, 42]. Overall, CT program demonstrated a significant improvement in the gait endurance (0.96 [95% CI: 0.46–1.46], *p* = 0.001), and moderate significant heterogeneity among the studies (I² = 74.03%, *p* = 0.002) (see Fig. 5). Both the visual assessment of funnel plots and the results from Egger’s test did not indicate any significant publication bias (*p* = 0.50). The trim-and-fill method identified two missing studies on the left side of the mean; however, the results remained unchanged after correction (0.62 [95% CI: 0.07–1.25]).

### Effect of combined training program on fatigue

**Fig. 6:**
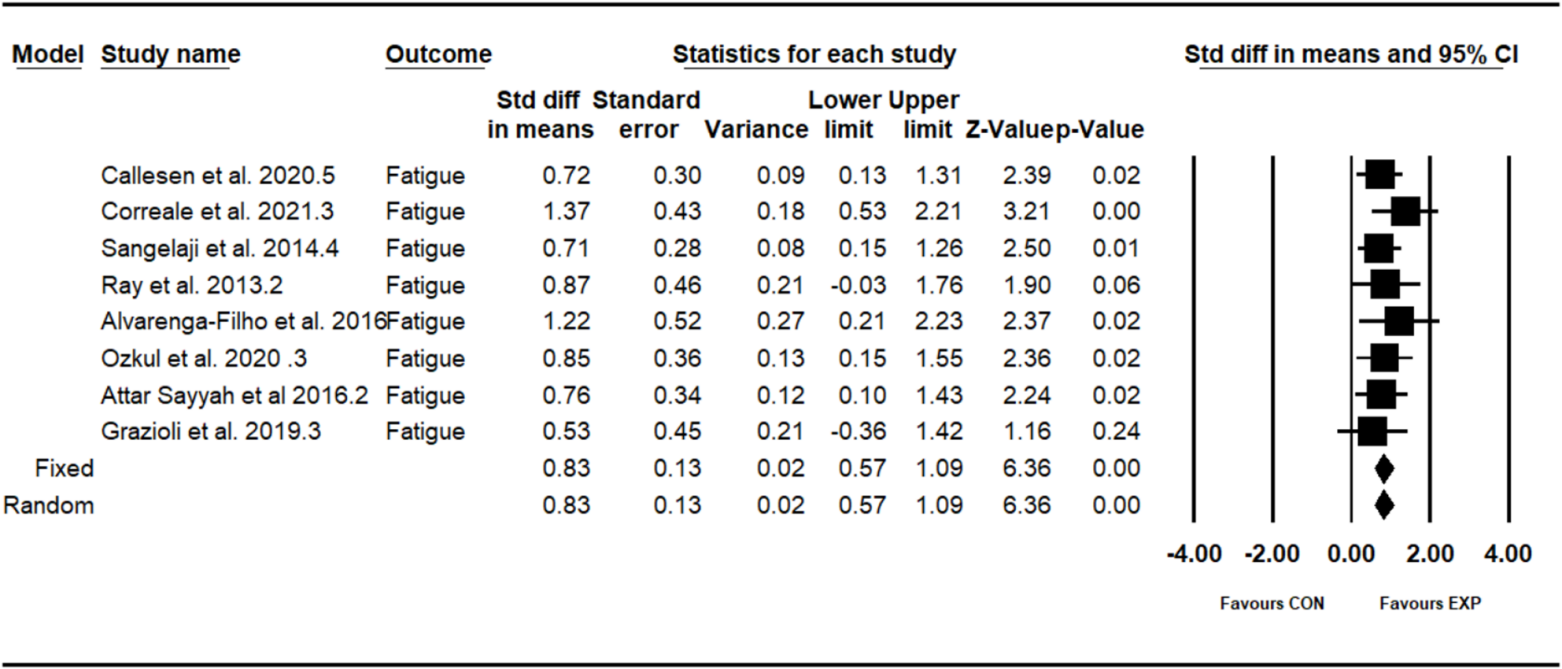
Forest plot of the combined training program versus control group analysis on fatigue

The analysis included eight intervention arms examining the effect of the CT program on the fatigue [12, 34, 39, 41–45]. Overall, CT program demonstrated a significant improvement in the fatigue (0.82 [95% CI: 0.57–1.08], *p* = 0.001), and no significant heterogeneity among the studies (I² = 00.00%, *p* = 0.88) (see Fig. 6). Both the visual assessment of funnel plots and the results from Egger’s test did not indicate any significant publication bias (*p* = 0.18).

### Effect of combined training program on quality of life

**Fig. 7:**
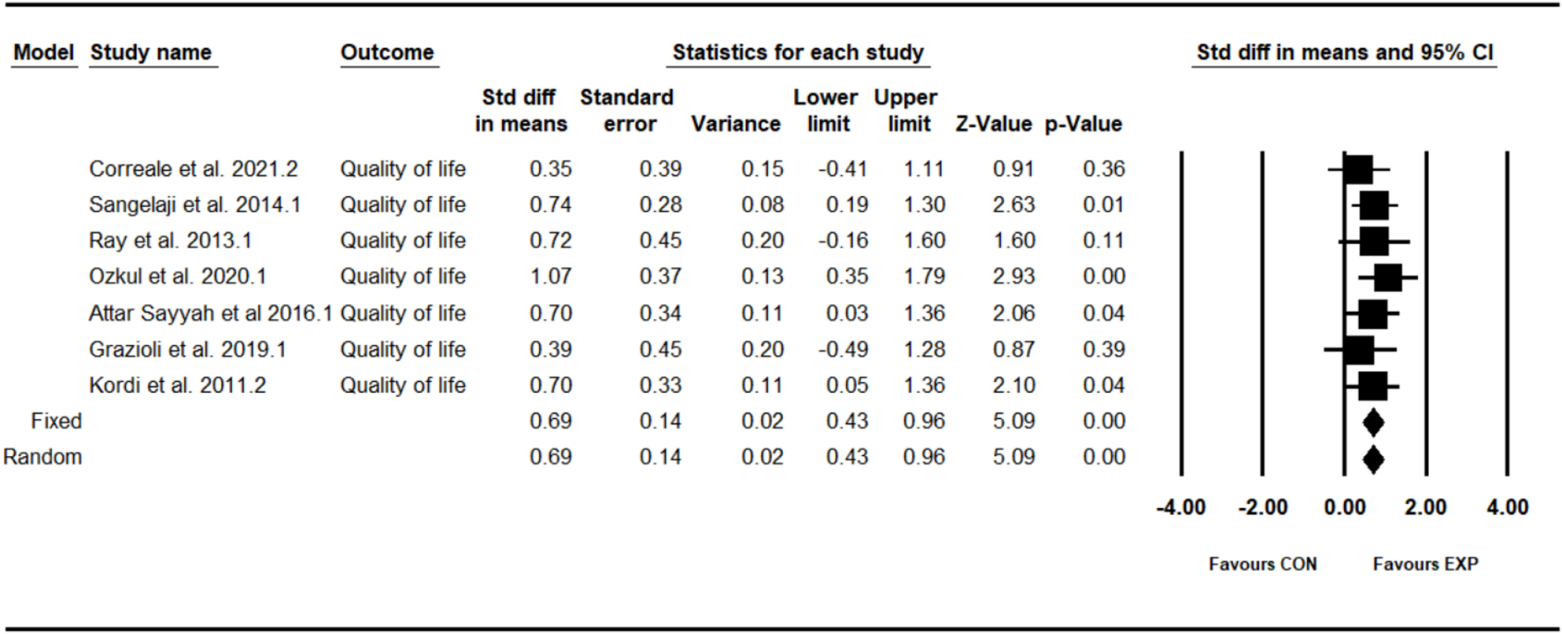
Forest plot of the combined training program versus control group analysis on quality of life

The analysis included seven intervention arms examining the effect of the CT program on the quality of life [34, 37, 39, 41–43, 45]. Overall, CT program demonstrated a significant improvement in the quality of life (0.69 [95% CI: 0.43–0.96], *p* = 0.001), and no significant heterogeneity among the studies (I² = 00.00%, *p* = 0.88) (see Fig. 7). Both the visual assessment of funnel plots and the results from Egger’s test did not indicate any significant publication bias (*p* = 0.44). The trim-and-fill method identified one missing study on the left side of the mean; however, the results remained unchanged after correction (0.63 [95% CI: 0.38–0.88]).

## Discussion

The current systematic review and meta-analysis aimed to assess the impact of CT on muscles strength, balance, gait speed, gait endurance, fatigue, and quality of life of PwMS. The results showed that CT has a positive effect on the muscles strength, balance, gait speed, gait endurance, and quality of life of PwMS. The results of the study also showed that CT reduced fatigue in PwMS. The results of this systematic review and meta-analysis are consistent with several other studies. One systematic review found that combined balance and strength training effectively improves postural balance in stroke survivors [21]. Another meta-analysis indicated that aerobic, resistance, and CT significantly enhance HRQOL in PwMS [13]. Additionally, a separate meta-analysis revealed that combined strength and endurance training effectively helps PwMS manage fatigue [22]. Together, these studies underscore the positive impact of CT on health outcomes for PwMS.

Moderate heterogeneity was observed in the outcomes related to muscle strength, balance, gait speed, and endurance. This variability is likely attributable to differences in intervention protocols, with durations ranging from 6 to 24 weeks (predominantly 8 to 12 weeks), frequencies of 2 to 3 sessions per week, and intensities varying from moderate (e.g., 60–80% of one-repetition maximum [1RM] in resistance training) to progressive (e.g., Borg scale ratings of 12–14 for aerobic components). Participant characteristics, particularly Expanded Disability Status Scale (EDSS) scores (mostly between 1 and 6.5), also played a role in this heterogeneity, with studies such as Sangelaji, Kordi [35] demonstrating larger effect sizes in milder cases. Despite this heterogeneity, random-effects modeling produced robust pooled estimates.

The quality of the studies, assessed using the PEDro scale, ranged from fair to high, with 50% of studies scoring between 7 and 11. Strengths were noted in aspects such as randomization and blinding; however, gaps in allocation concealment may have inflated effect sizes. The assessment of publication bias indicated low risk (Egger’s *p* > 0.1 for most analyses), although trim-and-fill adjustments modestly reduced SMDs, such as the balance outcome decreasing from 1.20 to 0.81, while still maintaining statistical significance. This supports the reliability of the results but cautions against generalizing findings to individuals with severe MS (EDSS > 6).

Combined training is defined as the integration of two exercise modalities. However, the interventions examined in various studies demonstrate considerable heterogeneity, such as combinations of resistance + aerobic, resistance + balance, and Pilates + aquatic exercise. This variability raises concerns about CT being a non-standardized concept. In reflecting on this study, it becomes evident that a clear delineation of the core components inherent in CT is crucial. These components endurance, strength, balance, and flexibility serve as the foundational elements that underpin CT. Systematically identifying and articulating these aspects enhances the evaluation of CT’s effects on health and fitness outcomes. Additionally, the incorporation of diverse exercise modalities not only bolsters the evidence supporting CT but also highlights its holistic nature in fostering improved physical fitness. This comprehensive approach contributes to more robust conclusions regarding the multifaceted benefits of combined training across various population.

The analysis of twelve intervention arms [12, 27–37] demonstrates that CT significantly enhances lower limb muscle strength in PwMS. This strength enhancement is likely due to improved connectivity and communication among motor neurons, leading to better synchronization and recruitment of motor units [46]. Such neural adaptations can increase the rate of force production and the capacity for sustained muscular force during maximal contractions [47]. Additionally, resistance training may further facilitate these neural changes, enhancing both the activation and synchronization of motor units [48]. Ultimately, these adaptations contribute to increased muscular strength and improved neuromuscular coordination, enhancing functional task performance [49].

Positive impacts of CT on balance were observed in thirteen intervention arms [12, 28, 30, 31, 33, 35–41]. Balance is the physical ability to perform daily activities such as standing and walking, which depends on the proper functioning of the cerebellum, middle ear, vision, touch, tendons and joints, muscles, and the ability to coordinate these factors [50]. In PwMS, balance disorder is directly affected by the destruction of cerebellar tissue, and its side effects include sensory disorders, extreme fatigue, decreased vision, and muscle weakness, especially in the lower limbs [51]. The increase in patients’ balance observed in this study can be partially attributed to the increase in the strength of the knee extensor muscles that was developed following the CT intervention [40]. This improvement in balance is particularly important because PwMS often exhibit delayed postural responses. These delayed responses are related to the demyelination of the posterior spinal cord, which leads to impaired postural control. Therefore, strengthening the knee extensor muscles through CT helps mitigate these balance issues by improving the body’s ability to respond to postural challenges more effectively [52]. The effect of the balance exercise intervention is at least partially related to the role of sensorimotor inputs in postural control. Motion sense, especially proprioception, is crucial for the effectiveness of both postural feedback and feedforward controls [33]. The various modalities of CT appear to play a role in strengthening neural signals, contributing to improved sensorimotor processing integration and the reorganization of neural networks. This enhancement ultimately leads to better postural control in PwMS. However, it is important to note that the extent of these effects may vary depending on the specific type of exercise included in the CT regimen [12]. Additionally, the manipulation of visual system inputs during the balance training interventions appears to be an important factor in the neuromuscular reorganization and improvement of postural control, as visual inputs play a key role in the performance of coordinated movements [39].

Improvements in gait speed [12, 27–30, 32, 33, 35, 36, 38, 41] and endurance [12, 27, 29, 31, 32, 35, 36, 38, 39, 41, 42] were demonstrated across eleven intervention arms. The mechanism underlying the improvements in gait speed and endurance can be attributed to the multifaceted physiological and neurological adaptations induced by the CT approach. The increase in lower limb muscle strength observed in PwMS following CT may be attributed to changes in the connectivity and communication between motor neurons. These neural changes can lead to enhanced synchronization and recruitment of a greater number of motor units, which can improve the rate of force production and the capacity to exert sustained muscular force [53]. Additionally, the resistance training exercises included in the CT interventions may have facilitated neural adaptations, such as increased motor unit activation and improved motor unit synchronization [35]. These neural changes can enhance both the quality and quantity of functional task performance, ultimately resulting in increased muscular strength and improved neuromuscular coordination, which are crucial for gait performance [12]. Furthermore, the positive effects of CT on balance in PwMS can also contribute to the observed improvements in gait endurance and speed. The increase in patients’ balance can be partially attributed to the increase in the strength of the knee extensor muscles, as well as the role of sensorimotor inputs in postural control [54]. The CT interventions seem to play a role in strengthening neural signals, which leads to improved sensorimotor processing integration and the reorganization of neural networks, ultimately enhancing postural control and gait function in PwMS [33].

Furthermore, the results indicate that CT has positive impacts on quality of life in PwMS, supported by seven intervention arms [34, 37, 39, 41–43, 45]. The mechanism by which CT can improve quality of life in PwMS can be scientifically justified as follows. The review indicates that CT, which incorporates both aerobic and resistance training, can lead to improvements in the physical and mental domains of quality of life in PwMS [13]. These improvements are likely mediated through several key physiological and neurological adaptations. Improved skeletal muscle function, enhanced neuromuscular coordination, and positive effects on mental health appear to be the key mechanisms underlying the beneficial impact of CT on quality of life [37]. By targeting both the physical and mental aspects of health, the CT interventions described in the review appear to have a more comprehensive and beneficial influence on the overall quality of life of PwMS compared to single-modality exercise programs [39].

The results of eight intervention arms [12, 34, 39, 41–45] showed positive impacts of CT on Fatigue in the PwMS. Fatigue is a prevalent and complex symptom experienced by PwMS, but its underlying pathophysiology remains incompletely understood. Potential contributing factors to increased fatigue in PwMS include alterations in skeletal muscle function, such as a reduction in type I muscle fibers, decreased oxidative capacity, and a predominance of anaerobic activity in extrafusal skeletal muscle fibers [55]. These pathophysiological changes may contribute to the increased fatigue observed in this population [56]. Rehabilitation-based interventions have demonstrated greater efficacy in managing fatigue compared to medication-based therapies [22]. Recent studies have investigated the relationship between fatigue and physical activity in PwMS, raising important considerations regarding this debilitating symptom [57]. The findings presented are consistent with other research, suggesting that CT program may help alleviate fatigue in PwMS.

In terms of clinical implications, the FITT principles offer a valuable framework for implementing CT in rehabilitation settings. Frequency of training sessions should be tailored to individual capabilities, potentially ranging from two to three times per week to optimize benefits while allowing for recovery. Intensity is another critical factor; moderate to vigorous intensity may be necessary to achieve significant improvements in strength and endurance, but it must be carefully monitored to ensure safety. Time refers to the duration of each session, which could vary but should generally aim for at least 30 min of combined aerobic and resistance training. Finally, the type of exercises should be diverse, incorporating both aerobic activities (such as walking or cycling) and resistance training focused on major muscle groups to maximize functional gains. The feasibility of implementing CT interventions in clinical practice is also a vital consideration. Many PwMS may face physical limitations or fatigue that could affect their ability to adhere to a training program. Therefore, it is essential to develop individualized exercise plans that consider each patient’s unique capabilities and preferences. Safety is paramount; clinicians should ensure that exercises are appropriate for the patient’s current level of function and that modifications are available to accommodate any exacerbations in symptoms. Adherence to exercise programs is often a significant challenge in rehabilitation settings. Strategies to enhance adherence may include providing education about the benefits of exercise, setting realistic goals, and incorporating social support mechanisms, such as group training sessions or partnerships with exercise buddies. Monitoring progress and celebrating small successes can also motivate patients to maintain their commitment to a CT regimen.

Clinically, CT proves feasible and safe, with high adherence (> 80% in most trials) and rare adverse events (e.g., mild soreness). Applying FITT principles from synthesized protocols: frequency 2–3 sessions/week, intensity moderate (60–80% 1RM or Borg 12–14), time 30–60 min/session, and type multimodal (e.g., resistance + aerobic for strength/fatigue; balance + functional for gait). Tailor to EDSS: milder PwMS (EDSS < 4) benefit from progressive intensities, while moderate cases (EDSS 4–6) favor supervised circuits.

### Limitations and future scope

The current study exhibits several methodological limitations that necessitate further examination. Firstly, while the overall level of evidence appears robust, the substantial heterogeneity observed in certain outcome measures calls for careful interpretation, restricting the generalization of results across different types of training interventions. Additionally, our analysis identified notable publication bias in several included studies. Although we attempted to address this issue using the trim-and-fill correction method, the limitations of the random-effects model particularly in the presence of heterogeneity may have inadvertently favored smaller studies, and funnel plot asymmetry may have skewed the results. Lastly, the systematic review’s search was limited to studies published in Persian and English, suggesting that future research should expand the language scope to include a wider range of languages. Overall, these methodological limitations highlight the necessity for ongoing research to bolster the scientific rigor and applicability of findings related to the effects of CT programs across diverse populations and outcomes.

## Conclusion

The current systematic review and meta-analysis suggest that CT interventions may have a positive impact on various physical and functional outcomes in PwMS. Specifically, the results indicate that CT is associated with improvements in muscle strength, balance, gait speed, gait endurance, and quality of life, while also contributing to a reduction in fatigue within this patient population. However, it is important to moderate the interpretation of these findings. While CT shows promising effects, heterogeneity among studies and potential biases must be considered. Therefore, further studies are warranted to fully elucidate the efficacy of CT as a rehabilitation and management strategy for individuals living with multiple sclerosis.

## Supplementary Information

Supplementary Material 1

Supplementary Material 2

Supplementary Material 3

## Data Availability

The datasets used and/or analyzed during the current study are available from the corresponding author on reasonable request.
