# Supplementary material for: Combined training improves functional capacity, fatigue, and quality of life in individuals with multiple sclerosis: a systematic review and meta-analysis
Source: BMC Neurol. 2025 Dec 3;26:17. doi: 10.1186/s12883-025-04548-z (PMC12781395; doi:10.1186/s12883-025-04548-z)
Supplement: Supplementary file 3 — Supplementary Material 3 [file 12883_2025_4548_MOESM3_ESM.docx]

**Supplemental Table.** PEDro Scale Ratings

| **PEDro**  **Score** | **Point Measures**  **and Variability** | **Between-Group**  **Comparisons** | **Intention-to-Treat**  **Analysis** | **Follow-Up** | **Blind**  **Assessor** | **Blind**  **Therapist** | **Blind**  **Subject** | **Groups**  **Similar At**  **Baseline** | **Concealed**  **Allocation** | **Random**  **Allocation** | **Eligibility**  **Criteria** | **Study** |
| --- | --- | --- | --- | --- | --- | --- | --- | --- | --- | --- | --- | --- |
| 7.11 | 1 | 1 | 1 | 0 | 0 | 0 | 1 | 1 | 0 | 1 | 1 | Abaspour et al. 2022 ^27^ |
| 6.11 | 0 | 1 | 1 | 0 | 0 | 0 | 0 | 1 | 1 | 1 | 1 | Monireh et al. 2013 ^28^ |
| 5.11 | 0 | 1 | 1 | 0 | 0 | 0 | 1 | 1 | 1 | 1 | 1 | Broekmans et al. 2011 ^29^ |
| 8.11 | 1 | 1 | 1 | 0 | 0 | 1 | 1 | 1 | 1 | 1 | 1 | Callesen et al. 2020 ^12^ |
| 6.11 | 0 | 1 | 1 | 0 | 0 | 0 | 1 | 1 | 1 | 1 | 1 | Eftekhari et al. 2012 ^30^ |
| 7.11 | 0 | 1 | 1 | 0 | 0 | 0 | 1 | 1 | 0 | 1 | 1 | Monireh et al. 2012 ^38^ |
| 8.11 | 1 | 1 | 1 | 0 | 0 | 0 | 1 | 1 | 1 | 1 | 1 | Haghighi et. 2023 ^31^ |
| 8.11 | 1 | 1 | 1 | 0 | 0 | 0 | 1 | 1 | 1 | 1 | 1 | Abbaspoor et al, 2020 ^32^ |
| 8.11 | 1 | 1 | 1 | 0 | 0 | 0 | 1 | 1 | 1 | 1 | 1 | Gutiérrez-Cruz et al. 2020 ^33^ |
| 7.11 | 1 | 1 | 1 | 0 | 0 | 0 | 0 | 1 | 1 | 1 | 1 | Correale et al. 2021 ^34^ |
| 6.11 | 0 | 1 | 1 | 0 | 0 | 0 | 1 | 1 | 0 | 1 | 1 | Sangelaji et al. 2016 ^35^ |
| 6.11 | 0 | 1 | 1 | 0 | 0 | 0 | 1 | 1 | 0 | 1 | 1 | Sangelaji et al. 2014 ^39^ |
| 5.11 | 0 | 1 | 1 | 0 | 0 | 0 | 0 | 0 | 1 | 1 | 1 | Ray et al. 2013 ^43^ |
| 6.11 | 0 | 1 | 1 | 0 | 0 | 0 | 1 | 1 | 0 | 1 | 1 | Alvarenga-Filho et al. 2016 ^44^ |
| 8.11 | 1 | 1 | 1 | 0 | 0 | 1 | 1 | 1 | 1 | 1 | 1 | Ozkul et al. 2020 ^42^ |
| 7.11 | 1 | 1 | 1 | 0 | 0 | 0 | 0 | 1 | 1 | 1 | 1 | Najafi et al. 2019 ^40^ |
| 6.11 | 0 | 1 | 1 | 0 | 0 | 0 | 0 | 1 | 1 | 1 | 1 | Attar Sayyah et al 2016 ^45^ |
| 7.11 | 1 | 1 | 1 | 0 | 0 | 0 | 0 | 1 | 1 | 1 | 1 | Grazioli et al. 2019 ^41^ |
| 6.11 | 0 | 1 | 1 | 0 | 0 | 0 | 0 | 1 | 1 | 1 | 1 | Sayyah et al 2016 ^36^ |
| 5.11 | 0 | 1 | 1 | 0 | 0 | 0 | 0 | 1 | 0 | 1 | 1 | Kordi et al. 2011 ^37^ |
